# Small Bowel Obstruction as an Initial Presentation of Mesenteric Follicular Lymphoma: Case Report and Literature Review

**DOI:** 10.7759/cureus.21566

**Published:** 2022-01-24

**Authors:** Kaku Kuroda, Amie Lucia

**Affiliations:** 1 Family Medicine, State University of New York Upstate Medical University, Syracuse, USA; 2 Surgery, State University of New York Upstate Medical University, Syracuse, USA

**Keywords:** small bowel obstruction, extranodal lymphomas, bilateral abdominal pain, mesenteric tumor, follicular lymphoma

## Abstract

Follicular lymphoma is the second most common type of B-cell non-Hodgkin lymphoma. It is known as one of the indolent lymphomas. Although some cases presenting with abdominal masses with or without symptoms have been reported, generally, primary extranodal follicular lymphoma is uncommon. Moreover, small bowel obstruction (SBO) as an initial presentation is extremely uncommon. We encountered a unique case of mesenteric follicular lymphoma that presented with SBO as the initial clinical presentation.

A 61-year-old male presented with a three-day history of abdominal pain and recurrent vomiting. Abdominopelvic computed tomography revealed air-fluid levels in multiple small bowel loops, with a transition point associated with a mass-like mesenteric abnormality. The small bowel was resected, and a wedge resection was performed to relieve the obstruction and make a diagnosis. Histopathology and immunohistochemical staining confirmed the diagnosis of follicular B-cell lymphoma.

Mesenteric lymphomas are less likely to present with SBO as the initial clinical presentation due to their extraluminal location. According to the literature review on the types of lymphomas that cause SBO, follicular lymphoma accounted for only 4.9% of lymphoma cases with SBO due to its indolent course.

## Introduction

Follicular lymphoma is the second most common type of B-cell non-Hodgkin lymphoma and accounts for approximately 20% of all non-Hodgkin lymphomas [1]. Follicular lymphoma is known as one of the indolent lymphomas. Patients with follicular lymphoma often present with painless peripheral lymphadenopathies, such as cervical, axillary, or inguinal lymphadenopathy [2]. Although there are some reported cases of abdominal masses with or without symptoms, generally, primary extranodal follicular lymphoma is uncommon [3]. Moreover, small bowel obstruction (SBO) as an initial presentation of mesenteric lymphoma is extremely uncommon, and the exact epidemiology of mesenteric lymphoma is unknown. Here, we report a unique case of mesenteric lymphoma that presented with SBO as the initial clinical presentation. Furthermore, to our knowledge, there is no report on the etiological basis of the types of lymphomas that cause SBO. Therefore, we present a literature review on the types of lymphomas that cause SBO.

## Case presentation

**Figure 1:**
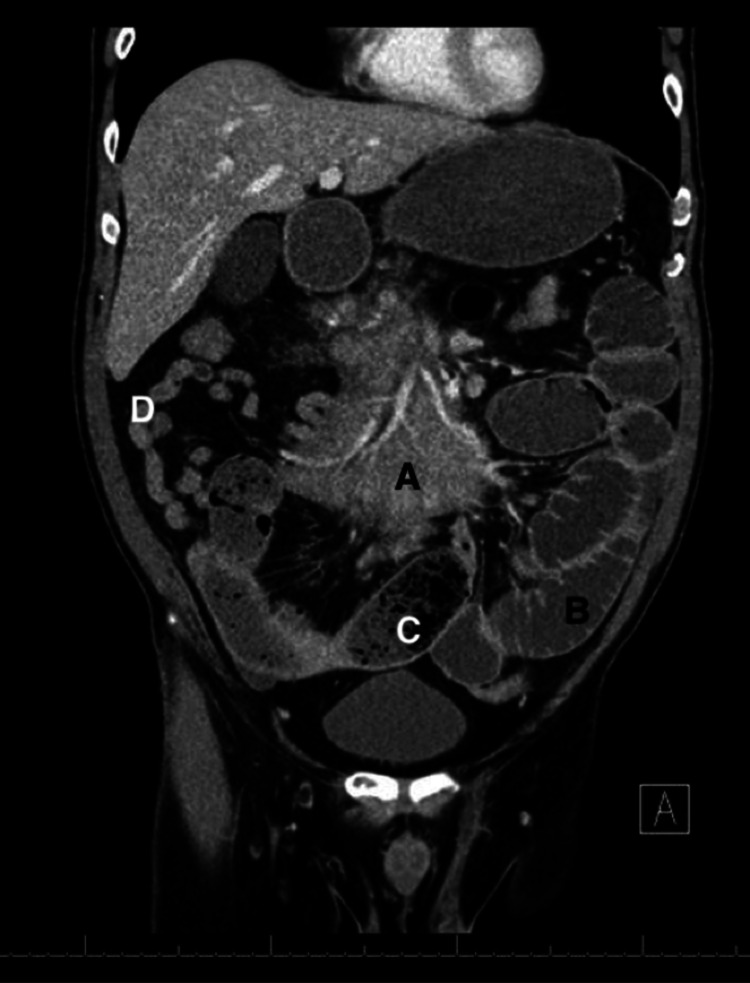
The coronal image of the contrast-enhanced computed tomography scan of the abdomen and pelvis. (A) Thickening of the small bowel mesentery consistent with a mass. (B) Dilated loops of the bowel with air-fluid levels. (C) Fecalization of the small bowel contents. (D) Collapsed loops of the distal small bowel.

**Figure 2:**
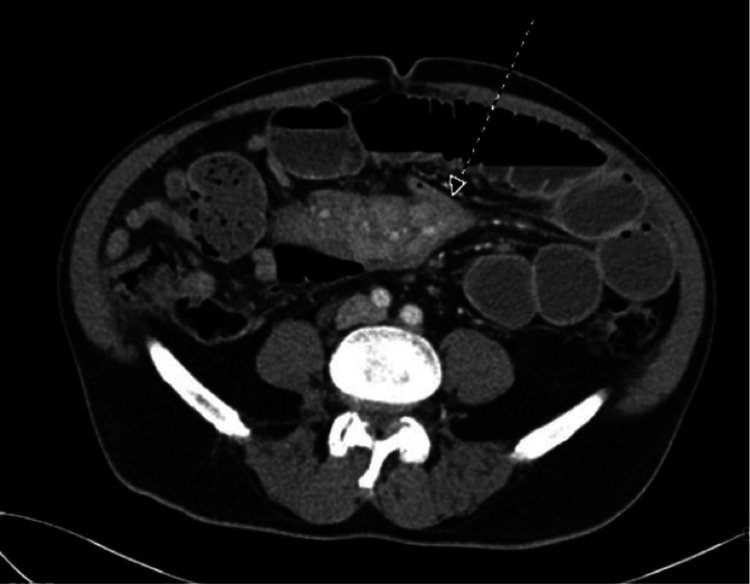
The axial image of the contrast-enhanced computed tomography scan of the abdomen and pelvis. The transverse section shows the transition point of the small bowel obstruction caused by the mesenteric tumor (arrow).

A 61-year-old male presented to the emergency department (ED) with a three-day history of abdominal pain and recurrent vomiting. He complained of gradual onset and progressive worsening of intermittent epigastric pain that had started after eating dinner three days prior to admission. Subsequently, he had several episodes of bilious vomiting. He stated that he had last passed stool and flatus the day prior to admission. His symptoms had persisted for three days despite a trial of oral anti-emetics at home. He had experienced similar but much less severe episodes in the past year. His medical history was significant for hypertension and paroxysmal atrial fibrillation. He had no surgical history. His medication included amlodipine. In the ED, his body temperature was 36.6°C, blood pressure was 132/85 mmHg, heart rate was 108 beats per minute, respiratory rate was 16 breaths per minute, and oxygen saturation was 96%. Physical examination revealed a distended abdomen that was tympanitic on percussion, with hypoactive bowel sounds and diffuse tenderness to palpation. No guarding or rebound tenderness was noted. Laboratory data revealed a white blood cell count of 3,900/mm^3^, a hemoglobin level of 15.2 g/dL, platelet count of 190,000/mm^3^, creatinine level of 1.0 mg/dL, aspartate aminotransferase level of 21 U/L, alanine transaminase level of 23 U/L, albumin level of 3.8 g/dL, and lactate level of 3.7 mmol/L. Contrast-enhanced computed tomography of the abdomen and pelvis revealed air-fluid levels in multiple small bowel loops (Figure 1), with a transition point associated with a mass-like mesenteric abnormality measuring 11.1 cm × 4.3 cm (Figure 2).

The patient was initially treated with volume resuscitation and gastrointestinal decompression with a nasogastric tube as the initial management of SBO. After hydration and decompression, exploratory laparoscopy was performed the following morning for both diagnostic purposes and surgical treatment. The patient was found to have thickened mesentery and a mass consistent with pathological central lymphadenopathy. There was a clear transition point where complete obstruction had occurred in the small bowel due to the mass on the mesenteric side. Based on the intraoperative findings, mesenteric lymphoma was suspected because the mass was originating from the mesentery and compressing the affected small bowel. The small bowel was resected, and a wedge resection was performed for the mesenteric mass to relieve the obstruction and make a diagnosis. Histological examination revealed large, dense follicles composed of small lymphoid cells, consistent with low-grade (1/3) follicular lymphoma. Immunohistochemical staining showed that the neoplastic cells were positive for CD20, pax5, CD10, BCL-2, and BCL-6 (focal), and negative for CD3, CD5, CD23, or BCL-1, confirming the diagnosis of follicular B-cell lymphoma. He was referred to the oncology department for further treatment. He received chemotherapy with bendamustine/rituximab and responded well to treatment. He is currently stable after six cycles of chemotherapy.

## Discussion

We encountered a unique case of mesenteric follicular lymphoma that presented with SBO as an initial clinical presentation. According to our literature review, follicular lymphoma is a rare cause of SBO due to its indolent course. This suggests that we can discuss the risk of SBO in patients with intestinal or mesenteric lymphomas depending on the type of lymphoma.

SBO is one of the important causes of surgical admission for acute non-traumatic abdominal pain. The most common etiology of SBO is adhesions within the abdomen or pelvis due to a history of intra-abdominal surgery [4]. Other major causes of SBO include complicated hernia, Crohn’s disease, and neoplasm [5]. In cases of malignant SBO, it has been reported that metastatic cancer is more common than primary tumors of the small intestine [4]. Primary tumors of the intestine that can result in SBO include small bowel adenocarcinoma, gastrointestinal stromal tumor, tumors of the cecum, and carcinoid tumor [6]. SBO due to primary mesenteric lymphoma, as discussed above, is not as common as SBO due to these malignancies. Although primary gastrointestinal tract lymphomas can cause endoluminal occlusion, mesenteric lymphomas are less likely to present with SBO as the initial presentation due to their extraluminal location [7].

In this case, histopathology confirmed the diagnosis of follicular lymphoma. However, it is unknown whether follicular lymphoma commonly causes SBO in adults, and what type of lymphoma causes SBO more frequently. Therefore, we conducted a literature review on the types of lymphomas that can cause SBO.

For our literature review, we performed a search in PubMed using the search terms “lymphoma, small bowel obstruction” and “lymphoma, intussusception.” We excluded articles that involved patients aged less than 19 years, reports on lymphomas in the large intestine, articles in non-English languages from which we could not determine the type of lymphoma being discussed, and articles with the diagnosis of lymphosarcoma because this term is no longer used. We found 82 cases of SBO including intussusception caused by lymphoma. Table 1 describes the types of lymphoma discussed in these articles.

**Table 1:** Description of the types of lymphomas that can cause small bowel obstruction. *Three cases were reported as plasmablastic lymphoma, one case as anaplastic lymphoma kinase-positive, large B-cell lymphoma, and one case as immunoblastic lymphoma associated with Crohn’s disease MALT: mucosa-associated lymphoid tissue

Type of lymphoma	N (%)
Diffuse large B-cell lymphoma*	35 (42.7)
Burkitt lymphoma	18 (22.0)
Mantle lymphoma	11 (13.4)
MALT lymphoma	5 (6.1)
Follicular lymphoma	4 (4.9)
Anaplastic large cell lymphoma	4 (4.9)
Enteropathy associated T-cell lymphoma	2 (2.4)
Post-transplantation lymphoproliferative disorders	2 (2.4)
Lymphoblastoma (Hodgkin’s type)	1 (1.2)

Among these articles, diffuse large B-cell lymphoma was the most common, followed by Burkitt lymphoma and mantle cell lymphoma. Follicular lymphoma accounted for only 4.9% of the cases of lymphoma with SBO, even though follicular lymphoma is the second most common type of lymphoma. We assume that these differences in the incidence of SBO are derived from the differences in the aggressiveness of these lymphomas. In general, diffuse large B-cell and Burkitt lymphomas are more aggressive, whereas follicular and mucosa-associated lymphoid tissue lymphomas are indolent [8]. Aggressive lymphomas present with more symptoms, including B symptoms (for example, fever, weight loss, and rapidly enlarging tumor masses), and can result in death within a few weeks if left untreated [9]. Extranodal disease, such as gastrointestinal lymphoma, is usually seen in aggressive lymphomas and is not common in indolent lymphomas [9]. It has been reported that follicular lymphoma accounts for 3.6% of all gastrointestinal lymphomas, which is thought to be one of the reasons why SBO as an initial presentation of follicular lymphoma is rare [10]. Additionally, SBO is an uncommon complication even in primary gastrointestinal follicular lymphoma [11].

The precise etiology of SBO due to lymphoma cannot be determined by our literature review because it is limited to only published articles. However, we believe that our review contributes to investigations of the tendency of developing SBO in both aggressive and indolent lymphomas. We hypothesize that SBO as an initial presentation of follicular lymphoma is rare compared to its incidence in aggressive lymphomas because of the differences described above.

## Conclusions

Mesenteric lymphoma presenting with SBO as the initial presentation is rare. When a patient without a surgical history presents with signs and symptoms consistent with SBO, it is crucial to investigate for intra and extraluminal malignancy. According to the literature review, the most common lymphoma that can cause SBO is diffuse large B-cell lymphoma, whereas follicular lymphoma is a rare cause of SBO due to its indolent course. This suggests that we can discuss the risk of SBO depending on the type of lymphoma, especially for intestinal or mesenteric lymphomas.
